# Alteration of barrier properties, stratum corneum ceramides and microbiome composition in response to lotion application on cosmetic dry skin

**DOI:** 10.1038/s41598-022-09231-8

**Published:** 2022-03-26

**Authors:** Barry Murphy, Sally Grimshaw, Michael Hoptroff, Sarah Paterson, David Arnold, Andrew Cawley, Suzanne E. Adams, Francesco Falciani, Tony Dadd, Richard Eccles, Alex Mitchell, William F. Lathrop, Diana Marrero, Galina Yarova, Ana Villa, John S. Bajor, Lin Feng, Dawn Mihalov, Andrew E. Mayes

**Affiliations:** 1 Unilever Research & Development, Port Sunlight, Bebington, Wirral CH63 3JW England, UK; 2 Institute of Infection, Veterinary, and Ecological Sciences, University of Liverpool, Liverpool, L69 7ZB England, UK; 3 Unilever Research & Development, Colworth, Bedfordshire, MK44 1LQ England, UK; 4 Unilever Research & Development, 55 Merritt Blvd, Trumbull, CT 06611 USA; 5 Eagle Genomics, Wellcome Genome Campus, Hinxton, Cambridge, CB10 1DR UK

**Keywords:** Microbiome, Microbiology techniques, Quality of life

## Abstract

Xerosis, commonly referred to as dry skin, is a common dermatological condition affecting almost a third of the population. Successful treatment of the condition traditionally involves the application of cosmetic products facilitating the moisturisation of the skin with a range of ingredients including glycerol and fatty acids. While the effectiveness of these treatments is not in question, limited information exists on the impact on the skin microbiome following use of these products and the improvement in skin hydration. Here, we describe improvements in skin barrier properties together with increased levels of cholesterol, ceramides and long-chain fatty acids following application of Body Lotion. Concomitant alterations in the skin microbiome are also seen via 16S rRNA metataxonomics, in combination with both traditional and novel informatics analysis. Following 5 weeks of lotion use, beneficial skin bacteria are increased, with improvements in microbiome functional potential, and increases in pathways associated with biosynthesis of multiple long chain fatty acids.

## Introduction

The human skin microbiome comprises the commensal microorganisms found on human skin, their combined genetic material and the environment in which they live^1^. The outermost skin layer, the stratum corneum, is composed of multiple layers of fully cornified keratinocytes embedded in a lipid matrix and crucially provides an environment for commensal skin microbiome colonisation^2^. Keratinocytes also play a key role in microbiome management effecting attributes such as pH, osmolarity and aridity^3^. Human skin can be divided into three location types based on physiological characteristics namely dry, sebaceous and moist sites. Each of these sites types harbour a distinct microbiome profile comprising organisms best suited to utilise the available nutrients in the specific niche^4^. Dry sites (leg or forearm) are commonly colonised by the most diverse range of bacteria from the Proteobacteria and Firmicutes phyla; sebaceous sites (face) by members of the *Cutibacterium* genus and moist sites (axilla), by *Staphylococcus* and *Corynebacterium* species^5^.

Skin microbiome composition has been shown to be relatively stable over time despite perturbations brought on by daily activities and hygiene practice^6^. However, more significant and prolonged dysbiotic states have been documented in multiple skin conditions including atopic dermatitis^7^, acne^8^ and dandruff^9^. Multiple studies have shown significant differences either in community composition between healthy and diseased states with treatment for the associated condition resulting in a renormalizing of community steady state^10^.

Xerosis, commonly referred to as dry skin, affects almost a third of the global population^11^. Characterised by rough or scaling skin, the prevalence of cosmetic dry skin has been associated with a number of factors including the environment, age, gender and genetic makeup^12^. Cosmetic dry skin can have a significant impact on quality of life, potentially leading to more serious conditions such as atopic dermatitis, facilitated by the penetration of allergens through a damaged epidermal barrier^13^. Studies have also demonstrated that barrier defects can lead to increases in systemic inflammation^14^.

Stratum corneum (SC) lipids play a crucial role in the permeation function of skin barrier, as well as many other aspects of health and disease^15^. The main lipid classes are ceramides, fatty acids and cholesterol, present in an approximately equal molar ratio^16,17^. These lipids form a highly-organised and densely-packed lamellar structure in the SC. There is significant heterogeneity within these lipids groups and especially within the ceramides with over 400 species and 12 subclasses having been identified in human SC. In xerosis, a strong correlation between dry skin attributes (conductance, dryness, roughness and scaliness) and the level of ceramides has been observed^18^. In atopic skin, decreased levels of ceramides and long-chain FFAs were observed with an increase of transepidermal water loss (TEWL)^19,20^. In addition to the decrement of ceramides, there is an apparent dysregulation of the ceramide profiles reported in atopic eczema (AE), with a significantly increased level of a total short-chain length of 34 carbon atoms^18^. The compositional chain length has also been associated with altered barrier function^15,21^. Treatment of xerosis regularly includes the use of humectants, occlusives or emollients to restore barrier integrity and improve the hydration of the SC, which in turn supports the establishment of a health-associated microbiome^2^.

Whereas assessment of the skin microbiome between health and disease states such as acne and atopic dermatitis are commonplace, almost no work exists examining the cosmetic dry skin microbiome and its response to treatment. This work investigates the impact of a marketed Body Lotion (BL) on both skin condition and the skin microbiome using a combination of qualitative and quantitative measures.

## Results

### Assessment of skin barrier properties

**Figure 1:**
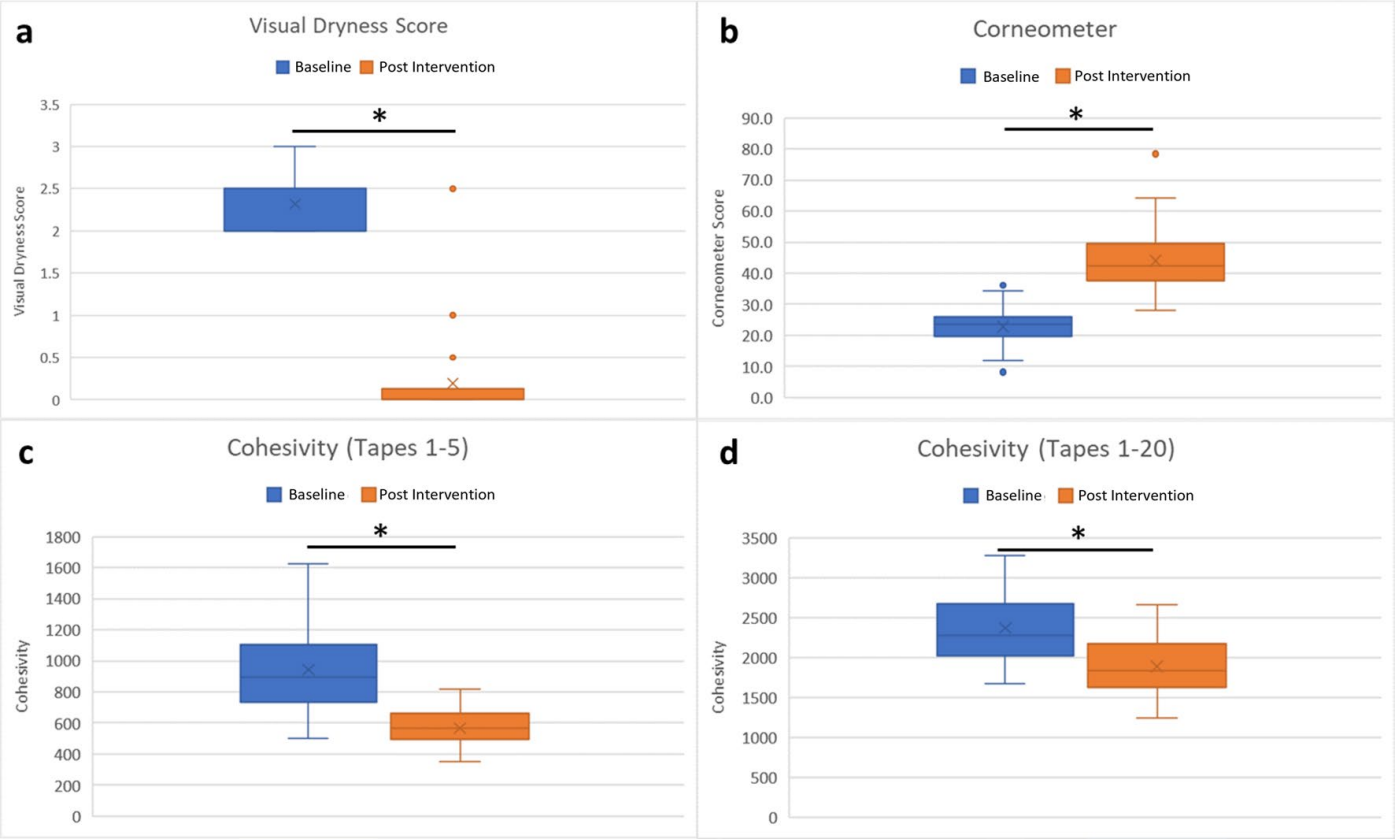
Lotion application improved leg skin hydration and cohesivity. Skin hydration was assessed at baseline and after 5 weeks of lotion application (n = 37) via (**a**) visual dryness and (**b**) corneometer. Stratum corneum cohesivity was assessed by protein removal on (**c**) 5 sequential tape strips and (**d**) 20 sequential tape strips (n = 36). Box whisker plots showing mean and upper and lower quartiles. Connecting line with an asterisk indicates a statistically significant difference.

Post intervention, visual assessment scores and corneometer measurements demonstrated significant improvements in hydration status (Fig. 1a,b). Mean visual dryness scores (with standard deviation) decreased from 2.3 (0.4) to 0.2 (0.2); whilst mean corneometer values increased from 22.6 (5.7) to 44.0 (10.2).

Stratum corneum cohesivity also improved with 5 weeks of product application with mean level of total protein (with standard deviation) removed by 5 sequential tape strips reducing from 944 (283 µg) to 569 µg (130 µg) (Fig. 1c). This increase in cohesivity was still evident following 20 sequential tape strips (Fig. 1d).

Samples were fractionated on amino-propyl solid phase extraction (SPE) columns to remove extracted adhesive material and partition the samples into cholesterol, ceramide and FFA fractions (see Supplementary Methods). FFA and Cholesterol measurements are quantitative. The amount of each analyte is back-calculated from the standard curve (has its own internal standard). Ceramide measurement is a semi-quantitative method due to its lack of the full set of the standards covering all ceramide classes and chain length.

Free fatty acid levels were measured in the outer-most 5 tape strips at both timepoints. Total FFA (with standard deviation) increased from 51.06 protein (25.63 pmol/µg) to 134.05 pmol/µg (68.07 pmol/µg), p-value < 0.001. Individual species analysis revealed significant increases in the levels of several FFAs from chain length C14:0 to C20:0 following the product application phase (Table 1). Levels of C22:0 also increased although the increase was not statistically significant, p-value = 0.086. Cholesterol (with standard deviation) was measured in the outer-most 5 tape strips at both timepoints with levels increasing from 29.85 pmol/µg protein (11.51 pmol/µg) to 32.35 pmol/µg protein (10.66 pmol/µg), p-value = 0.081. For depths of 16–20 tapes strips values at baseline were 40.94 pmol/µg (13.58 pmol/µg) rising post application to 63.34 pmol/µg (21.10 pmol/µg), p-value < 0.001.

**Table 1:** Levels of free fatty acids.

Fatty acid	Baseline average	Standard deviation	Week 5 average	Standard deviation	p-value
C14:0	2.63	2.54	4.99	3.07	< 0.001
C16:0	14.90	7.84	56.74	34.44	< 0.001
C16:1	1.73	1.81	1.77	1.22	0.316
C18:0	10.51	4.43	47.17	26.43	< 0.001
C18:1	8.46	8.28	9.20	8.60	0.184
C18:2	3.64	2.07	4.71	2.32	0.002
C20:0	0.67	0.16	1.47	0.64	< 0.001
C22:0	1.29	0.29	1.38	0.29	0.086
C22:1	0.00	0.00	0.03	0.03	0.44
C24:0	4.42	1.11	4.29	1.25	0.902

Average levels are given for each fatty acid species measured in pmol/µg. A mixed effects model was used to assess the differences between timepoints.

Average ceramides levels (with standard deviation) measured in 20 tape strips at baseline were 2.85 pmol/µg protein (0.70 pmol/µg) which rose to 3.80 pmol/µg protein (0.71 pmol/µg) post intervention—representing a 33% increase (p ≤ 0.0001). 358 ceramide species measured were assigned to their respective classes^22^ with class sum values calculated at both timepoints. For 9 of the 12 classes, statistically significant increases were seen in the levels post intervention (Table 2). The molar ratio among lipid groups of Cer:FFA:CHE is approximately 1:18:10 in our measures. The smaller amount of ceramide is due to the semi-quantitative approach and smaller sets of ceramides included in the calculation after a 70% occupational threshold described in the Supplementary Information.

**Table 2:** Levels of ceramide measured per ceramide class.

Ceramide class	Baseline average	Standard deviation	Week 5 average	Standard deviation	Fold change	p-value
AdS	0.23	0.07	0.31	0.09	1.36	0.003
AH	0.39	0.10	0.54	0.14	1.38	0.001
AP	0.25	0.09	0.35	0.12	1.42	0.01
AS	0.16	0.05	0.22	0.06	1.39	0.002
EOdS	0.00	0.00	0.00	0.00	1.26	0.68
EOH	0.17	0.06	0.25	0.09	1.53	< 0.001
EOP	0.02	0.01	0.02	0.01	0.97	0.71
EOS	0.23	0.08	0.28	0.09	1.22	0.03
NdS	0.30	0.07	0.36	0.07	1.2	0.06
NH	0.47	0.15	0.63	0.22	1.35	0.004
NP	0.35	0.10	0.45	0.10	1.27	0.024
NS	0.28	0.09	0.38	0.12	1.34	0.001

Average levels are given for each ceramide class in pmol/µg protein. Fold change was calculated for post intervention relative to baseline values with paired Wilcoxon testing used to assess the differences.

**Figure 2:**
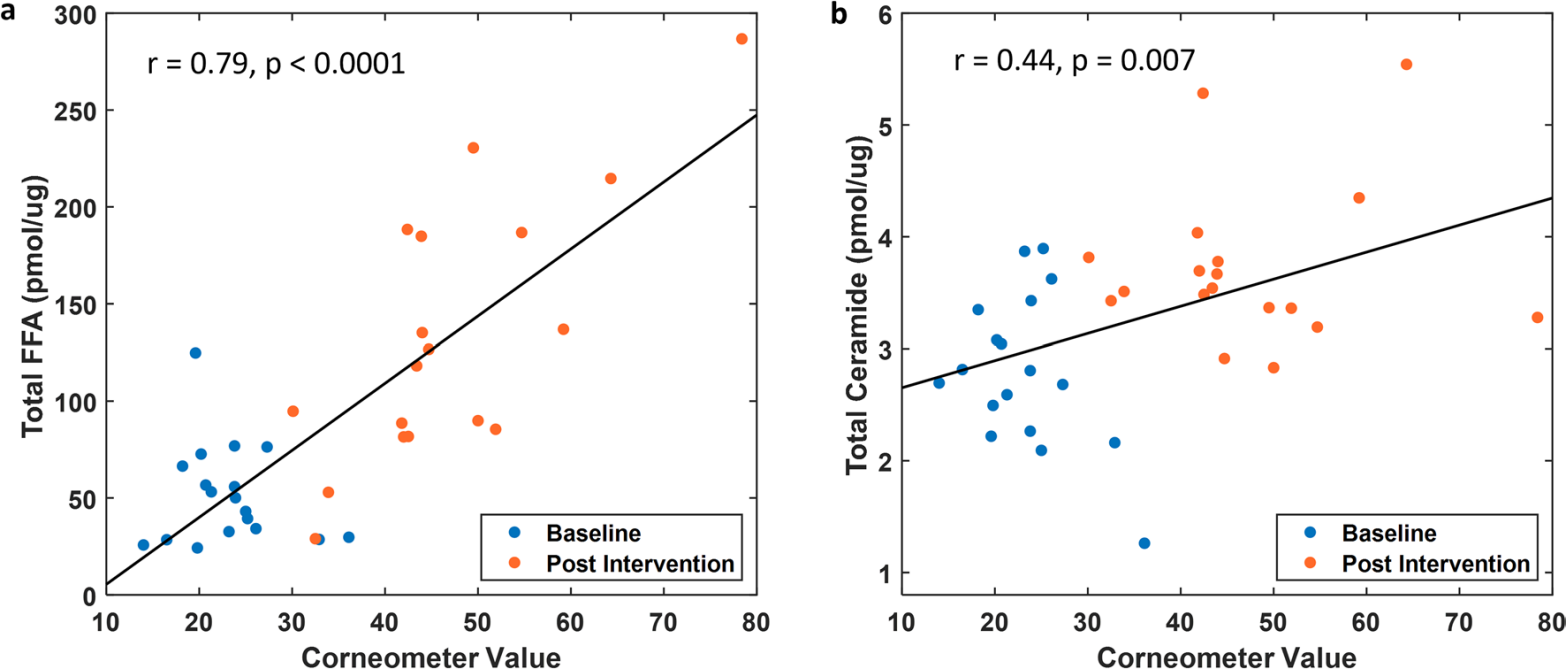
Skin hydration correlates with fatty acid and ceramide levels. Scatter plots of corneometer readings of skin hydration vs. the total level of FFA (**a**) and total ceramides (**b**). Blue and red circles represent untreated and after 5 weeks of product application. The solid line represents the optimal fit from simple linear regression analysis, with the correlation coefficient r and p-value displayed. Statistical significance was considered as p-value < 0.05.

Corneometer values of skin hydration were found to be positively associated with levels of both FFA (r = 0.79, p < 0.0001) and ceramides (r = 0.44, p = 0.007) (Fig. 2). Simple linear regression analysis was conducted to estimate the correlation and statistical significance. Pearson’s correlation method was used to calculate the correlation coefficient. Statistical significance was calculated by Student’s t-test.

### Microbiome assessment

**Figure 3:**
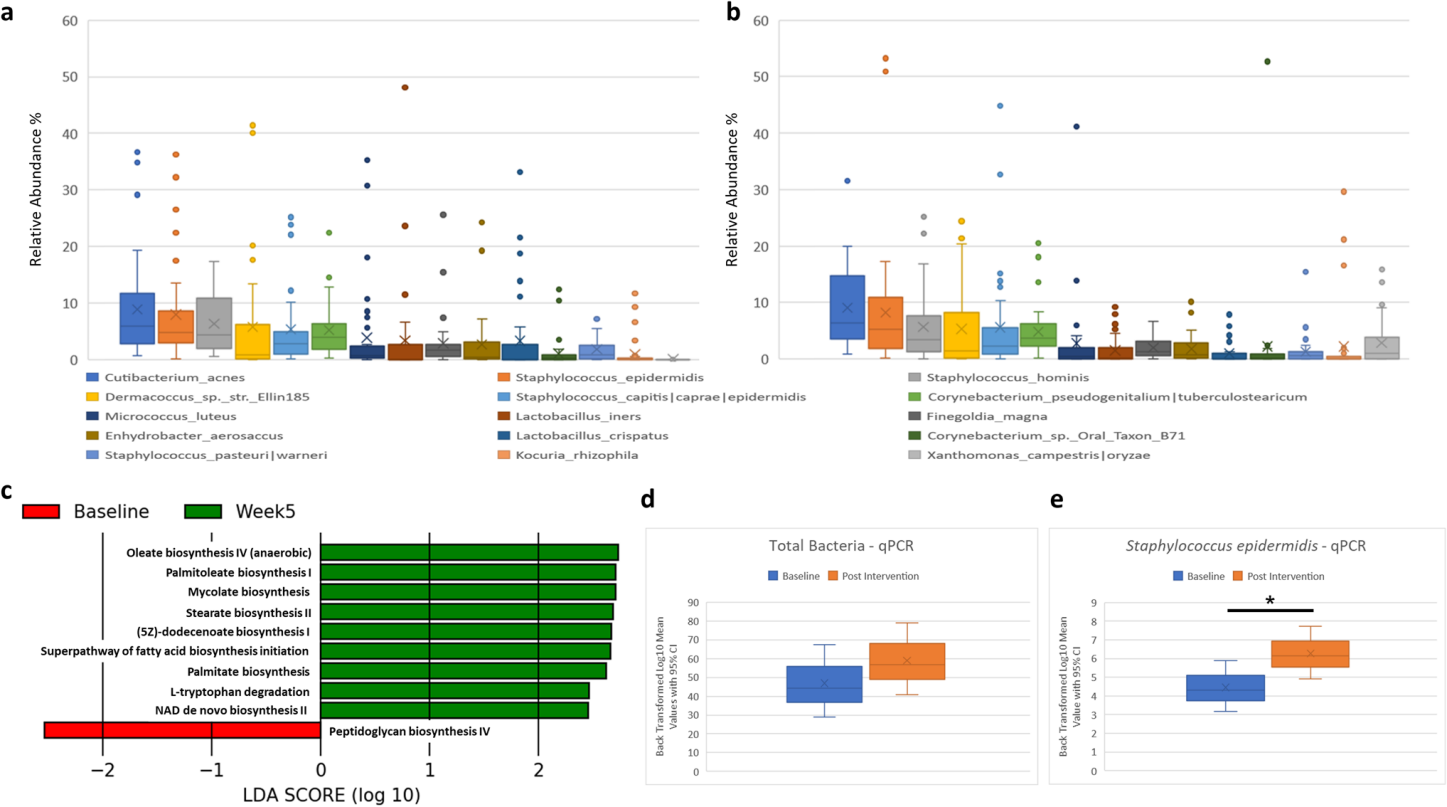
Skin microbiome assessment at baseline and after 5 weeks of product application. Box and whisker plots summarising the dominant species at (**a**) baseline and at (**b**) Week 5. (**c**) LEfSe analysis of differential microbiome function identified at baseline and Week 5. qPCR analysis of (**d**) total bacterial count and (**e**) *Staphylococcus epidermidis* at baseline and post intervention.

DNA sequencing of 70 samples (36 baseline and 34 post intervention) resulted in the generation of 22.8 million reads with an average read count of 326,333 reads per sample (min 63,892, max 975,103) Taxonomic assessment of the skin microbiome was visualized at Baseline (Fig. 3a) and Post Intervention (Fig. 3b).

At both timepoints dominant taxa were *Cutibacterium acnes*, *Staphylococcus epidermidis* and *Staphylococcus hominis*, aligning with published skin microbiome studies^4^. Differential abundance using ANCOM^23^ was carried out at both genus and species level at each timepoint. A single genus (*Xanthomonas*) and species (*Xanthomonas campestris*) were found to be more abundant in subjects post product application.

Samples were rarefied at 50,000 reads per sample in advance of diversity analysis. Alpha (Chao1 and Shannon) and Beta diversity (Unweighted and Weighted Unifrac)^24^ analyses were carried out to examine differences between baseline samples and following lotion use; however no statistically significant differences were seen (data not shown).

Microbiome functional profile were examined using Picrust2^25^ and statistical analysis carried out using LEfSe^26^, p-value 0.01 and LDA > 2. Nine functional pathways were shown to be elevated following product application in comparison to baseline (Fig. 3c). The majority of these pathways represented an increase in microbial production of skin-relevant fatty acids.

To quantitatively examine the impact of the intervention on akin bacteria the levels of total bacteria and the common health-associated commensal bacterium *S. epidermidis* were quantified using qPCR. No differences between timepoints were seen in the levels of total bacteria (Fig. 3d); however, a statistically significant increase (p < 0.01) was seen in the levels of *S. epidermidis* post intervention (Fig. 3e).

**Figure 4:**
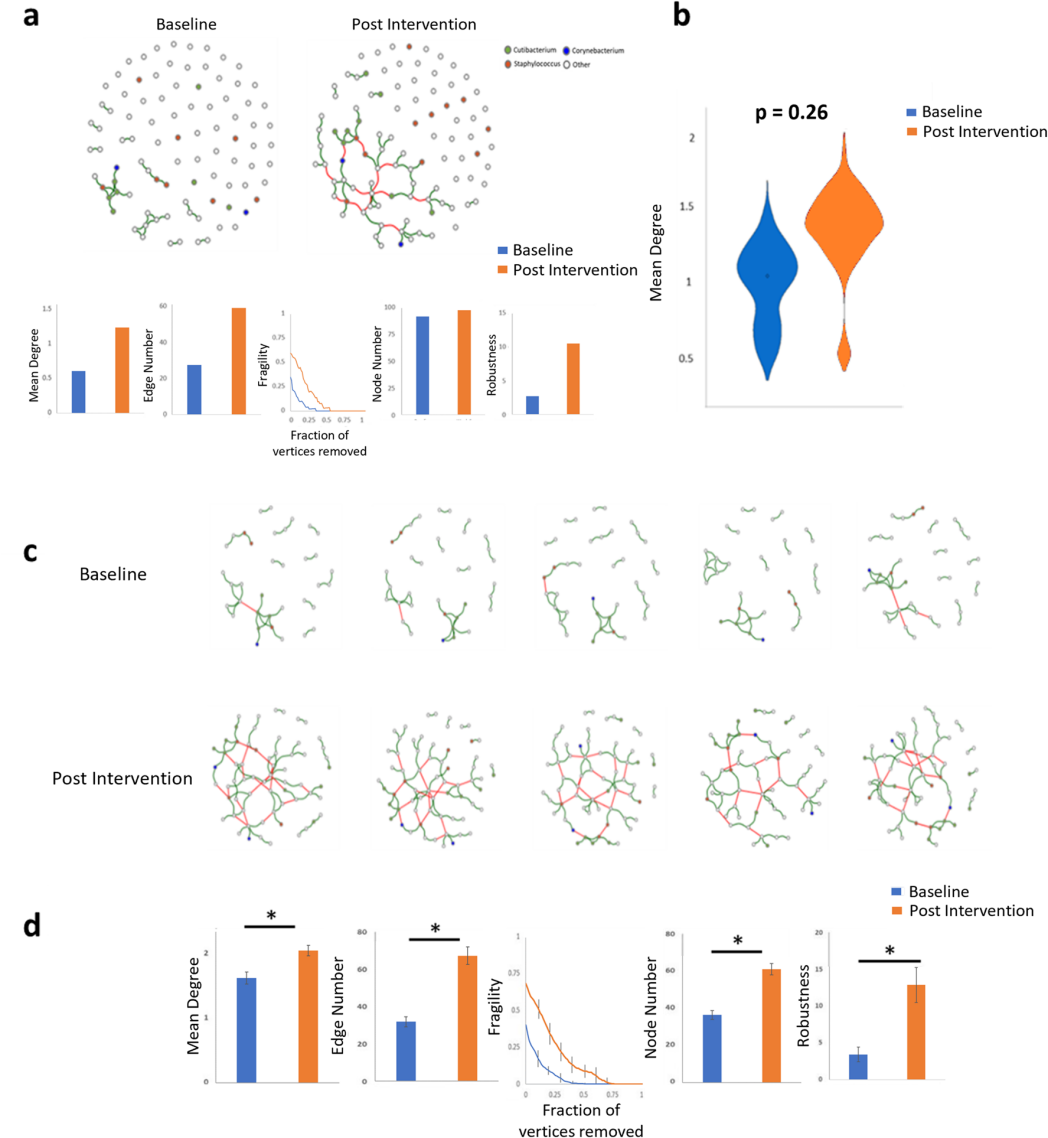
Community co-occurrence network analysis. (**a**) Average community networks for all samples at baseline and following 5 weeks of application, with associated network metrics. (**b**) Example statistical analysis of network mean degree metric following bootstrapping between samples at baseline and post intervention. No metrics used showed differences between pre- and post-application using average network approaches. (**c**) Selected indicative examples of single sample network from individual subject at baseline and post intervention. (**d**) Network analysis metrics between networks at baseline and post intervention.

Microbiome network of the population (MNP) for all samples at both timepoints was calculated. Analysis showed that network metrics including Mean Degree, Edge Number and Robustness were directionally increased post intervention (Fig. 4a). Additionally, networks from subjects after lotion use showed decreased fragility in comparison to networks at baseline. However, statistical analysis of these metrics using an 80% bootstrapping approach revealed no significant differences between networks when averaged across all samples at both timepoints (Fig. 4b).

Single sample networks were produced following methodologies described previously^27^. The production of n-1 networks facilitates the analysis of networks at the subject level and additionally allows improved statistical analysis of associated network metrics. Indicative examples of single sample networks can be seen in Fig. 4c. All single sample networks produced are available in Supplementary Material (Fig. S1). Statistical analysis of network metrics based on the aggregate single sample networks showed statistically significant differences for all metrics between baseline and post intervention, (p < 0.01) (Fig. 4d).

## Discussion

The aim of this study was to examine the impact on both host biomarkers and the skin microbiome following 5 weeks use of a marketed lotion containing glycerol, FFAs and a small amount of occlusives. Analysis of multiple skin biomarkers showed consistent improvement after the intervention period. Expertly measured visual skin dryness, an assessment of the impact on the upper layers of the stratum corneum, showed significant improvements for the study population. The impact however was not limited to the outermost levels of the skin since improved stratum corneum cohesivity was demonstrated even after 20 tape strippings. It should also be noted that improvements in skin condition were not only evident at the end of the 5 weeks of lotion use, as improvements in visual dryness were observed earlier. Indeed 86% of the study participants in this cell showed a significant improvement in skin dryness score after only 7 days of lotion use (data not shown).

The levels of SC lipids have been extensively linked to skin function^15^. In particular, lower levels of lipids, and especially longer chain lipids, have been reported in compromised skin^18,19^. In the current study, significant increases were seen in total ceramides, FFAs and cholesterol following body lotion application. These increases and their correlation to improvements in skin dryness provide further evidence for the critical role of lipids in maintaining good SC hydration and barrier function. While it is expected that some of the increase in the levels of C16:0 and C18:0 may be due to residual product deposition, the increase in longer chain fatty acids e.g., C20:0 and C22:0 suggests both increased synthesis and elongation from shorter chain precursors. It is also reasonable to assume that the sizable increases in C16:0 and C18:0 will provide the substrates for the sustained production of elongated species. Given their inherent heterogeneity within the SC, the increases seen in 9/12 classes of ceramide, together with the scale of those increases demonstrates wide-ranging improvements across the spectrum of these lipids. This is again consistent with improvements in SC hydration and barrier function and goes beyond the changes which might be achieved by topical supplementation with a small number of specific ceramide species. Whilst the changes in skin hydration may be attributable to the glycerol and occlusives present in the lotion tested^28^, the changes in lipid composition are more likely attributable to the free fatty acids provided. Studies have previously demonstrated the ability of ex vivo skin to produce greater levels of fatty acids (and in particular elongated species) when provided with shorter chain precursors^29^. The current study adds to this evidence by testing in vivo in human participants.

In addition to improvements in skin condition and composition, changes in the skin microbiome were also examined. Microbiome assessment of skin pre- and post-lotion use showed limited differences between the groups using standard analysis methods for the assessment of compositional metataxonomic data. No differences were seen in alpha and beta diversity metrics with only a single genus/species being differentially abundant, *Xanthomonas*/*X. campestris*, the increased abundance of which will be discussed later.

Metataxonomic assessment of the microbiome is limited in its ability to quantitatively access individual members of the microbiome as these methodologies only examine percentage compositional changes which can mask absolute abundance alterations. Based on its commonly accepted role as a beneficial skin bacterium^30^ the absolute abundance of *S. epidermidis* along with total bacterial abundance at baseline and after lotion use was examined to determine if there was an alteration in the number of bacteria present. While no differences were seen in total bacterial levels a significant increase was seen for *S. epidermidis* post intervention. Quantitative assessment of *Staphylococcus aureus* was also carried out on all samples however 95% of samples were below the limit of detection of the assay (data not shown). *S. epidermidis* is one of the most important members of the microbiome for maintenance of skin health^30^. *S. epidermidis* has been shown to activate skin TLR2 and TLR3 via the production of lipoteichoic acid stimulating the production of human β-defensins and inhibiting the inflammatory cytokine release and inflammation^31–33^. More recently a potential role for *S. epidermidis* in protecting against skin cancer via the production of 6-*N*-hydroxyaminopurine has been postulated; however further investigations in this area are warranted^34^. Indeed, recent activities have examined the beneficial impact of the application of autologous *S. epidermidis* to the skin of clinical subjects. Augmentation of resident populations with cultured *S. epidermidis* was shown to increase the lipid content of the skin, suppress water evaporation and helped to maintain the skins acidic pH through the production of lactic acid, a metabolic endpoint of glycerol fermentation^35^. Even more intriguingly, recent investigations into the actions of *S. epidermidis* on skin have demonstrated the presence of an active secreted sphingomyelinase in S. epidermidis clinical isolates potentially contributary to the release of free ceramides on skin^36^. The increase in *S. epidermidis* numbers following lotion use in this study is considered to be in line with an improvement in the condition of the underlining stratum corneum and its associated commensal microbiome.

Predictive functional analysis was carried out on all samples to determine changes in microbial functional profile following application. As discussed earlier some of the increases in skin lipids identified following product use are likely a consequence of product deposition however the increase in the bacterial potential to produce C16:0, C16:1 and C18:1 lipids, among others, present the intriguing proposition that the increase in skin lipids is at least partially derived from alterations in the functional output of the skin microbiome. Increased levels of bacterially derived oleate, palmitoleate, stearate and palmitate, all lipids with known beneficial functions on skin e.g., emollients, provide initial evidence that lotion use not only affects the skin barrier directly but also can potentially alter the skin microbiome to a state which may provide additional skin care benefits.

Co-occurrence network analysis has become a common analysis tool for assessment of the human microbiome^37,38^. Standard network analysis of study groups indicated no significant differences between subjects following lotion use. Standard network analysis approaches, where all subjects are collapsed into predetermined groups, suffers from significant limitations as it does not account for individual response to interventions. It is well documented that a high level of inter-individual variation occurs in the human skin microbiome in particular with regards to less dominant community members^4^. Recently, methodologies have been developed to examine microbiome networks at an individual level to facilitate the examination of an individual’s response to intervention^27^. Examination of single sample networks showed that all study subjects showed improvement in commonly used network analysis metrics including mean degree, edge number, node number and network robustness. Increases in these metrics are generally considered to indicate a more robust and interconnected microbiome network consistent with a healthier state. Based on these metrics a more health associated microbiome was present following the product intervention in line with the aforementioned improvement in key skin biomarkers.

Finally, we sought to address the increase in the levels of *X. campestris* following lotion use. An increase in the abundance of *Xanthomonas* following use of cosmetic products has previously being reported^39–41^ however bacteria of the genus *Xanthomonas* have rarely been reported in non-intervention-based studies of the skin microbiome at significant levels. Analysis of the test product showed the presence of xanthan gum, a common cosmetic ingredient to stabilise emulsions. Xanthan gum is produced by fermentation of monosaccharides by *Xanthomonas campestris*. None of the steps in the current production process would be sufficient to remove DNA from the producing organism during the purification process^42^. Subsequent analysis showed that both the xanthan gum and body lotion used in this study contained DNA that mapped to *X. campestris* (> 95% of reads generated, data not shown) suggesting this as the source of the increase, as opposed to any change in the skin microbiome as previously reported. These findings highlight the need to consider all potential sources of contamination in particular when assessing low biomass samples and an unexpected increase in an organism not commonly regarded as a skin commensal.

Due to the natural variation found in the skin microbiome which can be impacted by gender, age, body site and environmental conditions the study population was limited to pre-menopausal female subjects between the ages of 18–55. Subsequent studies could examine the impact of lotion application on a wider age cohort and include both male and female subjects and sample from alternative body sites. Examination of dose response is also feasible to examine the impact on skin parameters and microbiome compositions with differing concentrations of active ingredients.

This study set out to investigate the impact of moisturising lotion use on both skin health and microbiome structure and function in subjects with cosmetic dry skin. Our results show that BL provided increases in SC lipid content and cohesivity, with associated improvements in visual dryness and skin hydration. The impact of emollient use has been examined for a number of conditions including atopic skin^43,44^ and diaper dermatitis^45^. Studies, in diseased, atopic, populations show that emollient use reduces the levels of *S. aureus* with subsequent increases in alpha diversity and in some cases increases in the relative abundance of *S. epidermidis*. However, no information was previously available on the impact of a moisturizing lotion on the skin microbiome in subjects with cosmetic dry skin, a non-diseased population, where *S. aureus* is not correlated with the condition. Our data demonstrate that the composition, metabolic potential, and interconnectivity of the microbiome are improved following lotion use. This included quantitative increases in the known beneficial skin bacterium, *S. epidermidis*. This is the first demonstration of such an effect on cosmetically dry skin as a result of moisturiser application and suggests that improvements in skin barrier function may be, in part, mediated by alterations in the skin microbiome composition, function, and connectivity.

## Materials and methods

### Ethics statement

Written informed consent was obtained from all enrolled individuals. The study protocol was approved by the Reading Independent Ethics Committee. Methods were carried out in accordance with the principles of the Declaration of Helsinki and Good Clinical Practice as applicable to clinical studies on cosmetics.

### Study participants

Female subjects were recruited following appropriate inclusion and exclusion criteria including being aged 18–55 years with moderate levels of skin dryness on both lower outer legs. A full list of inclusion and exclusion criteria can be seen in Supplementary Tables 1 and 2. Skin sites were graded based on the dryness scale found in Supplementary Table 3. Subjects enrolled in the study had scores between 2.0 and 3.0 on the dryness scale and ≤ 1.0 on the erythema scale.

### Study design

**Figure 5:**
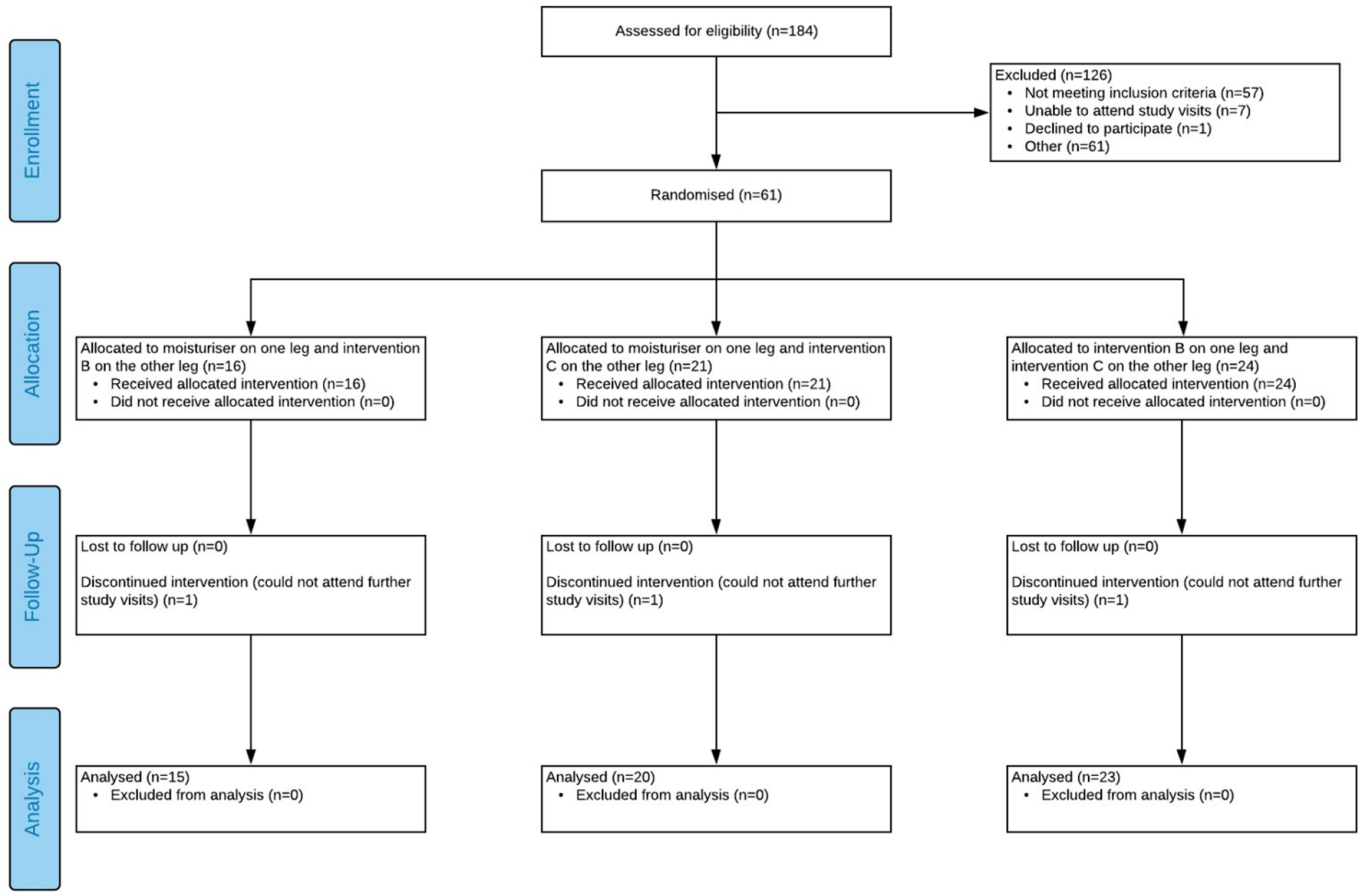
CONSORT 2010 flow diagram. Application of lotion application to legs of subjects classified as having cosmetic dry skin.

This was a single centre, 5-week, balanced incomplete-block design, lower-leg study. Three cells were investigated in this study, as outlined in Fig. 5. Only the data generated for one of the cells, body lotion, are discussed here. Prior to any skin assessments subjects were instructed to not apply any products, including study lotion, to their lower legs for 48 h. On the day of measurements, subjects were equilibrated in a temperature and humidity-controlled room (temperature 20 °C, relative humidity 50%) for 15 min ahead of assessments and sampling. Baseline measurements of skin dryness were conducted, followed by sampling of the skin microbiome and the collection of tape strips. Subjects were asked to apply BL twice daily to one lower leg for 5 weeks after which subjects were resampled. All samples were taken approximately 48 h post the last application of lotion.

### Skin dryness assessment

Visual assessment of skin dryness and erythema was conducted by a trained evaluator on a scale from 0 to 4^46^. Thirty seven subjects with visual dryness of 2.0–3.0 and erythema of ≥ 1.0 at the baseline visit were enrolled in the study. Corneometer assessments were taken, after an acclimation period of 15 min in an environmentally controlled room using the Courage and Khazaka Multi-Probe Adaptor Corneometer (MPA 6). Five readings were taken at each test site at both study timepoints.

### Stratum corneum cohesivity assessment

Stratum corneum cohesivity was assessed as described previously^47^ for all study subjects. D-Squame tapes (D100, 22 mm diameter, Clinical and Derm LLC) were applied to the designated area, pressed firmly onto the skin for 2 s with a D-Squame Pressure Instrument (Clinical and Derm D-500) to ensure constant pressure for each tape, then removed using forceps. Sequential tapes were taken from the same site then stored at − 20 °C. Total proteins were extracted from the tapes^47^ and assayed using the Pierce BCA assay kit (Rockford, IL, USA).

### Sample collection and extraction of stratum corneum lipids

Stratum corneum lipids were analysed from a population subset (n = 18) at both study timepoints. Cholesterol, ceramides, and FFAs were measured from samples collected using Leukoflex tape strips (2.5 cm × 8 cm) (BSN Medical, UK). Up to 20 sequential tapes were collected from each site and stored at − 20 °C prior to extraction. All tapes were collected, stored and extracted individually. FFA and Cholesterol analyses were performed on pooled sets of 5 tapes, whist ceramide analyses were performed on a pooled sample from all 20 tapes taken at a specific site. Detailed methods for the extraction, and quantification of FFA, cholesterol and ceramides can be found in Supplementary Information.

### Microbiome sample collection and processing

Buffer washes were collected from all participants using a sterile Teflon sampling ring using the cup scrub method^48^ as previously described^9^. Details of DNA extraction, library preparation, sequencing, informatics processing and qPCR are outlined in Supplementary Information.

### Microbiome network of the population (MNP) analysis

Co-occurrence network analysis was carried out using the Microbiome Network of the Population (MNP) method on QIIME2 generated ASV tables collapsed at species level. Taxa were filtered based on 0.01% relative abundance and 37% prevalence thresholds. Networks were inferred using Sparse InversE Covariance estimation for Ecological Association and Statistical Inference (SPIEC-EASI version 1.0.7)^49^. The neighbourhood method was chosen^50^ and the StARS (Stability Approach to Regularization Selection) method^51^ used with a lambda max threshold of 0.01.

Reproducibility of the network inference was performed using randomly resampled subsets. Sample subsets were resampled at the 80% level and used to generate 1000 networks. Mean values of the network connectivity and robustness features from the networks were compared using bootstrap hypothesis testing^52^.

### Microbiome network of an individual (MNI) analysis

Networks for individual samples within the population were calculated using the microbiome network of an individual (MNI) method^53^. Networks generated via this method with an individual sample removed, are subtracted from the MNPs of the whole sample set, resulting in a network specific for that sample.

### Network connectivity and robustness calculations

Network connectivity measures were calculated using the R package igraph (version 1.2.4.1)^54^. Network fragility was simulated by removing nodes sequentially and calculating the remaining size of the largest connected component^55,56^. Nodes were removed based on decreasing betweenness centrality and node degree and the stability assessed by natural connectivity; the average eigenvalue of the graph adjacency as the graph reduces in size. The robustness score was calculated as the Area Under the Curve (AUC) of the network fragility values.

### Statistical analysis

A detailed description of the statistical methods used for clinical measures, microbiome analyses, qPCR, ceramides and fatty acid analysis can be found in Supplementary Material.

## Supplementary Information

Supplementary Information.

## Data Availability

Datasets related to this article can be found in the SRA (Accession Number PRJNA701447).
