# Supplementary material for: Alteration of barrier properties, stratum corneum ceramides and microbiome composition in response to lotion application on cosmetic dry skin
Source: Sci Rep. 2022 Mar 26;12:5223. doi: 10.1038/s41598-022-09231-8 (PMC8957616; doi:10.1038/s41598-022-09231-8)
Supplement: Supplementary file 1 — Supplementary Information. [file 41598_2022_9231_MOESM1_ESM.docx]

**Title:** Alteration of Barrier Properties, Stratum Corneum Ceramides and Microbiome Composition in Response to Lotion Application on Cosmetic Dry Skin

**Short Title:** Dry skin and its microbiome following lotion application.

Barry Murphy^1*^, Sally Grimshaw^1^, Michael Hoptroff^1^, Sarah Paterson^1^, David Arnold^1^, Andrew Cawley^1^, Suzanne E Adams^1^, Francesco Falciani^2^, Tony Dadd^3^, Richard Eccles^2^, Alex Mitchel^5^, William F. Lathrop^4^, Diana Marrero^4^, Galina Yarova^4^, Ana Villa^4^, John S. Bajor^4^, Lin Feng^4^, Dawn Mihalov^4^, Andrew E Mayes^3^.

^1^Unilever Research & Development, Port Sunlight, Bebington, Wirral, England, CH63 3JW, United Kingdom

^2^Institute of Infection, Veterinary, and Ecological Sciences, University of Liverpool, Liverpool, England, L69 7ZB, United Kingdom

^3^Unilever Research & Development, Colworth, Bedfordshire, England MK44 1LQ, United Kingdom

^4^Unilever Research & Development, 55 Merritt Blvd, Trumbull, CT 06611, United States

^5^Eagle Genomics, Wellcome Genome Campus, Hinxton, Cambridge CB10 1DR, United Kingdom

**Supplementary Information**

**Supplementary Table 1:** Key Inclusion Criteria

| **Criteria** | **Description** |
| --- | --- |
| I1 | 18 to 55 years, |
| I2 | Female, |
| I3 | Fitzpatrick skin type I-III; |
| I4 | BMI 18.5-39.9 (inclusive) |
| I5 | Have the required visual assessment criteria on both lower outer legs at the beginning of phase 1 (2.0-3.0 dryness and ≤ 1.0 erythema) on 0-4 Dryness and Erythema Grading Scale with difference between top and bottom halves of lower legs and between right and left sides of the body ≤ 1.0 unit). |
| I6 | Willing and able to read and sign an Informed Consent Form, comply with the study restrictions, and carry out all study procedures. |

**Supplementary Table 2:** Key Exclusion Criteria

| **Criteria** | **Description** |
| --- | --- |
| E1 | Is menopausal, post-menopausal or using any form of HRT |
| E2 | Has a history of serious illness that may require regular systemic medication (e.g. thyroid dysfunction, liver dysfunction) which may influence the study outcome (in the opinion of the study doctor). |
| E3 | History of any type of cancer (if have had cancer must be greater than 6 months remission) |
| E4 | Has any immunological disorder (including, but not limited to, hepatitis, HIV+, AIDS, systemic lupus erythematosus, rheumatoid arthritis, hepatitis) |
| E5 | History of any dermatological condition on the leg in adult life; e.g. eczema, psoriasis, ichthyosis, atopic dermatitis |
| E6 | Is diabetic |
| E7 | Use of immunosuppressive drugs; e.g. methotrexate, cyclosporine, prednisolone for the two months prior to the start of the study or if has started taking these medications during the study |
| E8 | Use of antimicrobial drugs; e.g. penicillin, cephalosporins, tetracyclins, fusidic acid for the two months prior to the start of the study. |
| E9 | Use of any lipid lowering medication (e.g. statins) for two months prior to the start of the study. |
| E10 | Use of any steroidal medicine (inhaled, oral or topical) |
| E11 | Use of any topical medication on the lower outer leg |
| E12 | Use of systemic anti-inflammatory medication on a frequent basis (at the discretion of the medical monitor) |
| E13 | Has suspected allergy to soap, shower gel, moisturising products or any product containing yeast as well as any alcohols (such as rubbing alcohol or isopropanol) |
| E14 | Has excessive erythema or excoriations on the lower leg |
| E15 | Has test sites with tattoo, scars or any other features that may affect the study procedures. |
| E16 | Is currently pregnant or breast feeding (based on self- report only), or has given birth to or breast fed a child within the last 12 months. |
| E17 | Use of a sunbed or sun-shower in the month prior to the start of the study, or using one during the study or planning a holiday in the sun during the study. |
| E18 | Currently suffering from any problems with the back, hips or legs that would make sampling from the lower outer leg excessively uncomfortable for the subject or logistically difficult for the study team. |
| E19 | Has any allergy to plasters/adhesive tape |
| E20 | Smokers |

**Supplementary table 3:** Erythema and Dryness Scoring scale

| **Grade** | **Erythema Scale** | **Dryness Scale** |
| --- | --- | --- |
| 0.0 | No erythema | No dryness |
| 0.5 |  | Perceptible dryness, fine white lines |
| 1.0 | Mild erythema | Fine dry lines, white powdery look and/or some uplifting flakes, on less than 30% of the test site |
| 1.5 |  | A more uniform flaking, covering 30-50% of the test site |
| 2.0 | Moderate confluent erythema | Uniform, marked flaking covering more than 50% of the test site area and/or isolated scaling |
| 2.5 |  | Slight to moderate scaling |
| 3.0 ^a, b^ | Marked erythema | Moderate to severe scaling with some uplifting of the scales |
| 3.5 ^b^ |  | Severe scaling and/or slight fissuring |
| 4.0 ^b^ | Deep erythema | Severe scaling and severe fissuring |
| a No further product/assessments will be performed on any test site receiving a dryness and/or erythema score of > 3.5.  Other test sites receiving dryness and erythema scores of < 3.0 will continue to receive product/assessments. b Skin responses reaching a grade >3.5 for dryness or erythema and skin responses reaching grade 3.0 for dryness or erythema that increases in severity are considered adverse events | | |

**Supplementary Table 4:** QIIME2 Software Parameters

| **Software Parameter** | **Value** |
| --- | --- |
| FastQC filtering min gc percentage | 25 |
| FastQC filtering max gc percentage | 75 |
| FastQC filtering min average base quality | 20 |
| DADA2 forward read trim position | 21 |
| DADA2 reverse read trim position | 19 |
| DADA2 forward read truncation position | No truncation |
| DADA2 reverse read truncation position | No truncation |
| BLAST+ penalty | -5 |
| BLAST+ reward | 4 |
| BLAST+ gapopen | 5 |
| BLAST+ gapextend | 5 |

**Supplementary table 5:** Software Versions

| **Software** | **Version** |
| --- | --- |
| FastQC: | v0.11.5 |
| MultiQC | 1.7 |
| QIIME2 | 2019.4 |
| DADA2 | QIIME2 2019.1 |
| BLAST+ | 2.6.0+ |
| scikit-learn | QIIME2 2019.1 |


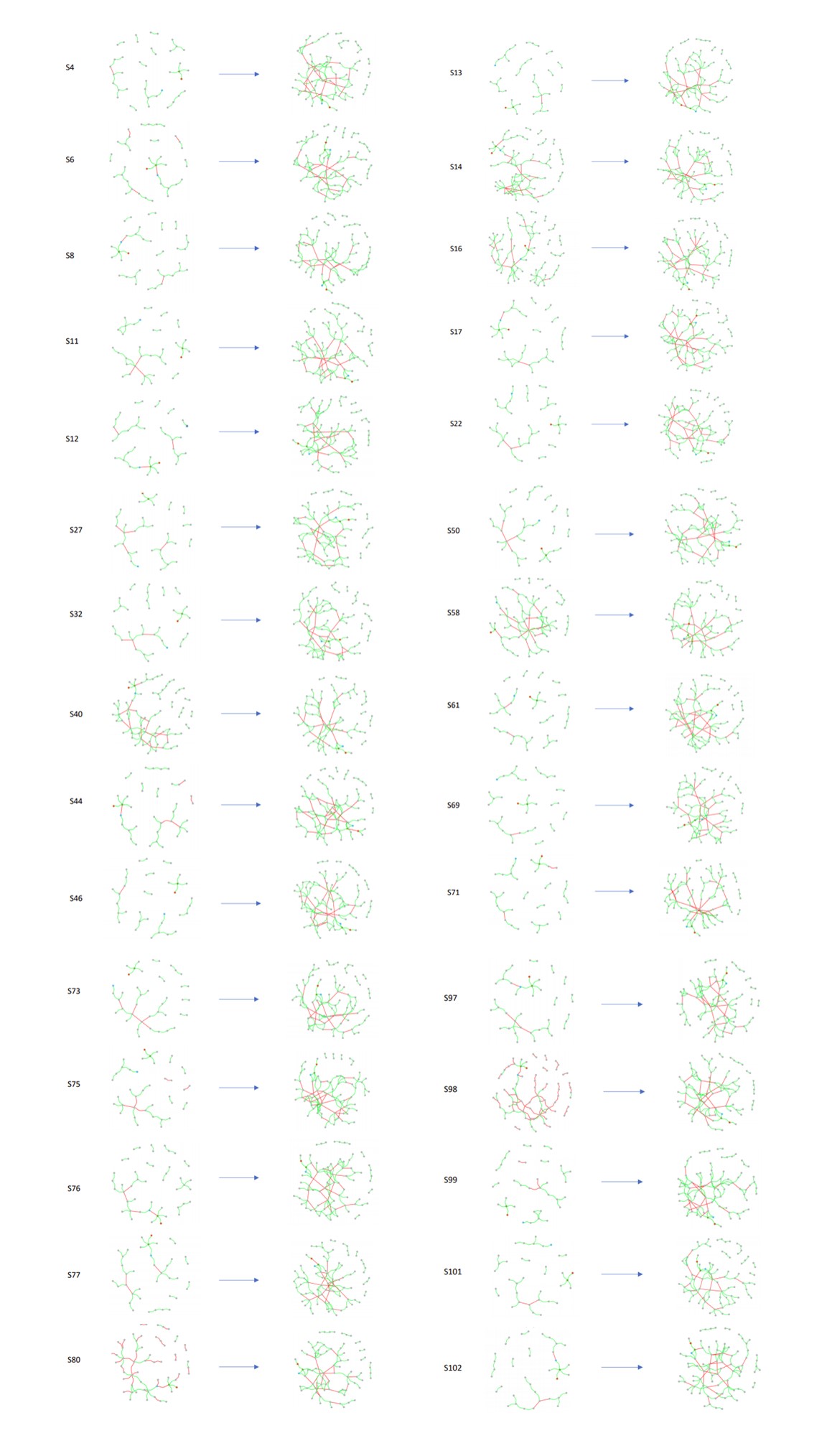


**Figure S1. Single Sample Network Analysis pre and post treatment.** Single sample network analysis of all samples at baseline and following lotion application for 5 weeks.

**Supplementary table 6:** Body Lotion Ingredients

| **Ingredient Class** | **Inclusion Level (%)** |
| --- | --- |
| Water | 77.62 |
| Glycerol | 10.00 |
| Surfactants | 6.55 |
| Occlusives / emollients | 3.75 |
| Sensory Modifiers | 0.5 |
| Structurants | 0.53 |
| Preservatives | 0.87 |
| Fragrance | 0.18 |

**Supplementary Methods:**

**Collection and extraction of Stratum Corneum lipids**

Lipid extraction was done in (80:20) methanol (MeOH): ethyl acetate (EtOAc), 10ml per tape at RT for 3 hours and solvent transferred to fresh tubes. Fifty percent of the extract from multiple tape strips combined, and the solvent evaporated to generate pooled lipid samples. Samples were fractionated on amino-propyl solid phase extraction (SPE) columns to remove extracted adhesive material and partition the samples into cholesterol, ceramide and FFA fractions. The cholesterol fraction was eluted with hexane (HX): ethyl acetate (85:15); the ceramide fraction was eluted with (66:33) chloroform (CHCl3): isopropanol (IPA); the FFA fraction was eluted with MeOH + 2% acetic acid. Extracted tape strips were incubated in 100mM sodium hydroxide (NaOH) at 60^o^C for 4 hours and the protein amount determined using the Pierce BCA kit.

**Detection and quantitation of free fatty acids (FFA)**

Stock solutions of all native fatty acid standards were prepared in CHCl3: MeOH at 1 mg/ml. Standard stock solutions were diluted with IPA/H_2_O for LC/MS analysis, to produce calibration standards in the range of 0.01 µg/mL to 7.5 µg/ml. Analyses were performed using a Shimadzu Prominence UFLCXR System (Shimadzu Precision Instruments Torrance, CA) connected to a SCIEX API 4000 mass-spectrometer (SCIEX, Redwood City, CA). Samples were separated on a reversed phase Halo C8 analytical column, 2.1x150 mm, particle size 2.7 µm (MACMOD Analytical, Chadds Ford, PA). Gradient elution was applied, starting at 100% mobile phase A (70:15:15 MeOH: Acetonitrile (ACN): H_2_O w. 0.1 % Ammonium acetate (NH4Ac)) toward 100% mobile phase B (75:25:5 IPA:ACN:MeOH w. 0.1% NH_4_Ac), holding for four minutes and returning to the initial condition of 100% mobile phase A. Flow rate was 0.3 ml/min. Injection volume was 2 µl. Total run time was 15 minutes. MS/MS measurements were performed using negative electrospray ionization mode (ESI), utilizing multiple reaction monitoring (MRM) method to selectively detect and quantitate native and deuterated fatty acid molecules. The method was set to detect ten native and seven deuterated fatty acids in clinical skin extracts. The acquisition method parameters are shown in the table below. For quantitation of native fatty acids, the following deuterated fatty acid standards were added to the processed clinical samples as internal standards before LC/MS analyses: d31-C16:0; d3-C22:0; d4-C26:0; 13C-18:1; 13C-18:2. The rest of deuterated fatty acids listed were added to select SPE cartridges during solid phase extraction of skin extracts to assess the extraction recovery. In this method, the parent-to-parent mass-to-charge (m/z) values were used in place of the parent-to-daughter m/z values, due to inability of FFAs to form daughter ions. This approach allowed for reduction of noise and increased accuracy of detection.

**Detection and quantitation of Cholesterol**

Stock solutions for Cholesterol and Cholesterol-d6 standards were prepared in CHCl3 at 1mg/ml. Standard stock solutions were diluted with combination of mobile phase A and mobile phase B for LC/MS analysis, to produce calibration standards in the range of 0.25 µg/ml to 5 µg/mL for Cholesterol and in the range of 0.063 µg/ml to 1 µg/mL for Cholesterol-d6. Analyses were performed using an I-class Acquity UPLC connected to a Xevo TQ-S mass-spectrometer (Waters, Milford, MA). Samples were separated on a reversed phase Phenomenex Kinetex EVO C18, 150 x 2.1 mm,1.7 u analytical column (Phenomenex Inc, Torrance, CA). Isocratic elution was applied, at 10:90 mobile phase A (ACN)/mobile phase B (70:30 ACN/MeOH) ratio. Flow rate was 0.2 ml/min. Injection volume was 2 µl. Run time was 4 minutes. MS measurements were performed using positive atmospheric pressure chemical ionization mode (APCI), utilizing selected ion recording (SIR) method to detect and quantitate molecular ions of Cholesterol and Cholesterol-d6 and to construct calibration curves. LC/MS acetonitrile and methanol OmniSolv® were purchased from EMD Millipore Corp (Burlington, MA). Chloroform HPLC plus grade was purchased from Sigma-Aldrich (St.Louis, MO). Cholesterol (≥99% purity) was purchased from Sigma-Aldrich (St.Louis, MO). Cholesterol-d6 (98% atom D) was purchased from CDN Isotopes (Pointe-Claire, Quebec, Canada).

**Detection and quantitation of Ceramides**

Ceramide analysis was performed via Lipotype Shotgun Skin Lipidomics method as described previously^1^. Data were analysed with in-house developed lipid identification software based on LipidXplorer^2^. Data post-processing and normalisation were performed using an in-house developed data management system. Only lipid identifications with a signal-to-noise ratio > 5, and a signal intensity 5‘-fold higher than in corresponding blank samples were considered for further data analysis. Data were analysed with R version 3.5.2 (2018-12-20)^3^ using tidyverse packages (version 1.2.1)^4^ and Bioconductor pcaMethods. A 70% occupational threshold has been applied to the data, i.e., only lipids which occur in at least 70% of the samples in either cohort are retained in the final dataset. This resulted in 358 lipids being included in the analyses.

**DNA Extraction**

Samples were defrosted and concentrated by centrifugation (10mins/13,000rpm, Eppendorf 5810R, Germany), supernatant removed, and the cells resuspended in 500 µl of sterile TE buffer (10 mM Tris-HCl; 1 mM EDTA, pH 7.4). The cell suspension was transferred to a 96-well Lysing Matrix Plate B (MP Biomedicals). Addition of 3 µl of Ready-Lyse lysozyme (Epicentre, 250 U/µl) was followed by incubation with agitation at 300rpm, 37^o^C for 18 hours. Following incubation, a bead-beating step was performed using a Tissue Lyser (Qiagen, Germany) for 3 minutes at 20 Hz. An off-board lysis was performed by incubating the samples at 68 °C for 15 minutes in the presence of Proteinase K, Carrier RNA, ATL and ACL buffer in a Qiagen S-plate following manufacturer guidelines. Post-incubation, the plate was loaded on to the QIAsymphony and the samples processed using the QIAsymphony Virus/Bact Midi Kit (931055, Qiagen). Extracted DNA was frozen prior to 16S rRNA library preparation.

**16S rRNA Library Preparation and Sequencing**

Oligonucleotide primers targeting the V1-V2 hypervariable region of the 16S rRNA gene were selected. PCR was carried out using the following primers,

U28F: 5'-ACACTCTTTCCCTACACGACGCTCTTCCGATCTNNNNNAGAGTTTGATCMTGGCTCA G-3’

U338R: 5'-GTGACTGGAGTTCAGACGTGTGCTCTTCCGATCTTGCTGCCTCCCGTAGGAGT-3’

PCR primers were modified version of the standard 28F and 338R primers which contain additional recognition sequences to facilitate nested PCR to add Illumina sequencing adapters and index sequences to resulting amplicons. PCRs consisted of 0.25 μl (10 μM) of each primer, 10 μl of HotStar Taq Plus Mastermix (Qiagen), 5 μl of template DNA and 4.5 μl molecular grade water (Ambion, Thermofisher). Samples were amplified using the following parameters: 95°C for 5 minutes, then 10 cycles of: 94°C for 45 seconds, 65°C for 30 seconds, and 72°C for 60 seconds, with a final extension of 10 minutes at 72°C using a Dyad Thermocycler (MJ Research). PCR products were purified using Ampure SPRI Beads (Beckman Coulter, California, USA).

A second round PCR incorporated Illumina adapters containing indexes (i5 and i7) for sample identification utilising eight forward primers and twelve reverse primers each of which contained a separate barcode allowing up to 96 different combinations.

General sequences of the primers are illustrated below with the variable 8 bp barcode underlined.

N501f 5′ AATGATACGGCGACCACCGAGATCTACAC*TAGATCGC*ACACTCTTTCCCTACACGACGCTC3′

N701r 5′ CAAGCAGAAGACGGCATACGAGAT*TCGCCTTA*GTGACTGGAGTTCAGACGTGTGCTC3′.

Second round PCRs consisted of 0.5 μl (10 μM) of each primer, 10 µl of 2 x Kapa Mastermix (Roche, Switzerland) and 9 μl of purified sample from the first PCR reaction. Samples were amplified using the following parameters: 98°C for 2 minutes, then 15 cycles of; 20 seconds at 95°C, 15 seconds at 65°C, 30 seconds at 70°C with a final extension of 5 minutes at 72°C. Samples were purified using Ampure SPRI Beads before being quantified using Qubit fluorimeter (Invitrogen, California, USA) and assessed using the Fragment Analyzer (Advanced Analytical Technologies, Iowa, USA). Resulting amplicon libraries were taken forward and pooled in equimolar amounts using the Qubit and Fragment Analyzer data and size selected on the Pippin prep (Sage Science, Massachusetts, USA) using a size range of 300–700 bps. The quantity and quality of each pool was assessed by Bioanalyzer (Agilent Technologies, California, USA) and subsequently by qPCR using the Illumina Library Quantification Kit (Kapa) on a Light Cycler LC480II according to manufacturer’s instructions (Roche, Switzerland). All libraries were sequenced on a flowcell of an Illumina HiSeq 2500 with 2 × 300 bp paired-end sequencing using v3 chemistry (Illumina, California, USA).

**Informatics Processing**

All steps were performed using the QIIME2 microbiome analysis tool suite^5^ version 2019.1. The paired end sequences were imported into QIIME2 format, then denoised using DADA2^6^. The primer sequence regions were removed during denoising by setting DADA2’s forward and reverse read trim parameters to the length of the forward and reverse primers, respectively. A complete list of software parameters and versions can be found in Supplementary Information Table 4 and 5. Denoising produced 13,715 unique amplicon sequence variants (ASVs) corresponding to 22,843,363 sequences in the 70 original samples. Samples were rarefied to 50,000 reads per sample in advance of diversity analysis. Rooted and unrooted phylogentic trees were generated for the ASVs using the QIIME2 phylogeny align-to-tree-mafft-fasttree workflow. Taxonomy assignments were generated by comparing ASVs against a BLAST database composed of the HOMD, HOMD extended and Greenegenes sequences (HOMDEXTGG version 14.51) described in^7^. Taxonomic classification was performed as previously described^8^ at 99 % identity across 98 % of the read length.

**Metagenomic Functional Prediction**

Functional profile prediction from 16S rRNA gene amplicon data was carried out using Phylogenetic Investigation of Communities by Reconstruction of Unobserved States 2 - PICRUSt2^9^. Gene family abundances were predicted based on ASV outputs from DADA2 using the default settings (picrust2_pipeline.py). Statistical analysis of function output was carried out using LEfSe^10^.

**qPCR Analysis**

qPCR quantification was performed for *S. epidermidis*, and for total skin bacteria. qPCR reactions for each target were set up individually in accordance with the following tables.

**Supplementary table 7**: qPCR reaction set up

| **Reaction mixtures** | **Volume (µl)** |
| --- | --- |
| DNA template (sample or plasmid standard) | 1 |
| Qiagen Probe PCR mastermix | 5 |
| Forward primer (10 μM) | 0.4 |
| Reverse primer (10 μM) | 0.4 |
| Probe (10 μM) | 0.2 |
| dd H2O | 3 |
| Total | 10 |

**Supplementary table 8:** Primer and probe sequences for each qPCR target.

| ***S. epidermidis*** |  |
| --- | --- |
| Forward primer | GGAGGAACTAATAATAAGTTAACTG |
| Reverse primer | GTCATAAACAGTTGTATATAAGCC |
| Probe | Fam-CTGCTAATCGTGGTGTTGCTCAAATTAAA-BHQ1 |
| **Total Bacteria** |  |
| Forward primer | AGAGTTTGATCMTGGCTCAG |
| Reverse primer | TGCTGCCTCCCGTAGGAGT |

qPCR reactions were carried out in triplicate on a RotorgeneQ qPCR system with the following cycling conditions,

Cycling-Program: 95°C 2min; (95°C 10 sec; 50°C 20 sec; 72°C 20sec) 45x; 4°C hold.

**Statistical Analysis**

To investigate the difference in visual dryness between baseline and week 5, a Wilcoxon Signed Rank test was applied: Change from baseline was calculated and analysed for difference to zero. To investigate the difference in instrumental hydration (Corneometer) and in skin cohesivity between baseline and week 5, paired t-tests were applied: change from baseline was calculated and analysed for difference to zero.

For free fatty acids and cholesterol, a mixed effect model was run with Change from Baseline (CFB) values based on Log10 transformed values as response variable, product as fixed effect, subject as random effect and Log10(baseline+1), age & BMI as covariates.

For ceramides, data were analysed with Lipotype in-house lipid identification software based on LipidXplorer^2^. Data post-processing and normalisation were performed using an in-house developed data management system. Only lipid identifications with a signal-to-noise ratio > 5, and a signal intensity 5‘-fold higher than in corresponding blank samples were considered for further data analysis. Data were analysed with R version 3.5.2 (2018-12-20)^3^ using tidyverse packages (version 1.2.1)^4^ and Bioconductor pcaMethods. Ceramides were measured in molar amounts and standardized to mass of protein. For ceramide class analysis, a 70% occupational threshold was applied to the data resulting in a dataset of 358 lipids to be compared. Class sums of individual lipid species are calculated with significance test via a paired Wilcoxon (Mann-Whitney) test.

Statistical analysis of 16S rRNA metataxonomic data including alpha and beta diversity analysis was carried out using the available scripts in QIIME2 version 2019.1. Differential abundance analysis was carried out using ANCOM^11^ in QIIME2 version 2019.1.

For qPCR data, log10 transformation was applied on raw values and paired t-tests used to compare the levels at baseline and after 5 weeks of lotion application.

**Supplementary REFERENCES**

1 Sadowski, T. *et al.* Large-scale human skin lipidomics by quantitative, high-throughput shotgun mass spectrometry. *Scientific Reports* **7**, 43761, doi:10.1038/srep43761 (2017).

2 Herzog, R. *et al.* LipidXplorer: a software for consensual cross-platform lipidomics. *PloS one* **7**, e29851-e29851, doi:10.1371/journal.pone.0029851 (2012).

3 R Development Core Team. R: A language and environment for statistical computing. R Foundation for Statistical Computing,. (2017).

4 Wickham, H. tidyverse: Easily Install and Load the 'Tidyverse'. (2017).

5 Bolyen, E. *et al.* QIIME 2: Reproducible, interactive, scalable, and extensible microbiome data science. *PeerJ Preprints* **6**, e27295v27292, doi:10.7287/peerj.preprints.27295v2 (2018).

6 Callahan, B. J. *et al.* DADA2: High-resolution sample inference from Illumina amplicon data. *Nat Methods* **13**, 581-583, doi:10.1038/nmeth.3869 (2016).

7 Al-Hebshi, N. N., Nasher, A. T., Idris, A. M. & Chen, T. Robust species taxonomy assignment algorithm for 16S rRNA NGS reads: application to oral carcinoma samples. *Journal of Oral Microbiology* **7**, 10.3402/jom.v3407.28934, doi:10.3402/jom.v7.28934 (2015).

8 Chen, T. *et al.* The Human Oral Microbiome Database: a web accessible resource for investigating oral microbe taxonomic and genomic information. *Database : the journal of biological databases and curation* **2010**, baq013 (2010).

9 Douglas, G. M. *et al.* PICRUSt2 for prediction of metagenome functions. *Nature Biotechnology* **38**, 685-688, doi:10.1038/s41587-020-0548-6 (2020).

10 Segata, N. *et al.* Metagenomic biomarker discovery and explanation. *Genome Biology* **12**, R60, doi:10.1186/gb-2011-12-6-r60 (2011).

11 Mandal, S. *et al.* Analysis of composition of microbiomes: a novel method for studying microbial composition. *Microbial ecology in health and disease* **26**, 27663, doi:10.3402/mehd.v26.27663 (2015).
